# Round the Hospitals

**Published:** 1916-07-01

**Authors:** 


### ROUND THE HOSPITALS.

PROGRESS is being made with the College of Nursing, and its successful establishment may be foreshadowed with hope, providing hurry is avoided and adequate time is given to solve a number of problems which offer mostly thistles and thorns at the start. The claim is being raised that every conceivable branch of nursing should not only be registered, but that each branch should have conferred upon it by Act of Parliament the right to appoint a representative on the College Council. Already the draft Bill recognises fever and mental nurses, and now a claim has been put forward for nurses who wish to specialise in children, in nervous, throat and ear, ophthalmic, orthopædic, and other cases, which threaten to so extend in variety and number as to make it possible that the only non-claimants may ultimately be members of the profession who wish to devote themselves exclusively to nursing patients with disease of, say, the little toe. Seriously, however, seeing that members of the medical profession specialise in an even larger number of departments, representing groups or a single group of diseases, and that they are content to be represented on the General Medical Council by members of the profession elected by the whole profession without group representation, it is evident that the formulation of innumerable claims by innumerable sections of the nursing profession to separate representation on the General Nursing Council and College exhibits a lack of apprehension rather than a just appreciation of the rules which govern State recognition by Act of Parliament. All sections of nurses who honestly desire State registration may have confidence that their just claims will be fully considered, and that they will receive adequate treatment by the Council of the College of Nursing, which, however, has to conform to precedent and practice when settling the clauses of the new Registration Bill.

---

The evolution of the College of Nursing, combined with a State Registration Bill, must put the whole nursing body throughout Great Britain to the test. Nurses may be enthusiastically in favour of State recognition, but if they are ever to secure it in the only form in which it can prove of immediate and increasing value, it is essential that each one of them should at the present stage of the negotiations exhibit intelligence, patience, a due recognition of the rights of other people, and a resolute discouragement of impossible claims. Further, it is essential that impulsive suggestions of the relative influence of individual representatives and of their power to secure or to stay a successful issue must be indignantly repudiated by all to whom they may be made. It is preposterous and wholly without reason or sufferance that any individual, whatever her position in the nursing world, by her actions should manœuvre for a position of predominant authority by working to put herself in the front and to belittle the knowledge, influence, and claims of her sister-matrons, ex-matrons, and fellow-nurses. State registration and the highest interests of the nursing profession must both suffer if such a policy is to be pursued or tolerated by anyone at the present juncture, when the search for a wise solution demands self-denial and straight conduct upon the part of all concerned. Thus only can a wise and unanimous settlement of the problems presented by the establishment of a General Nursing Council and the College of Nursing be secured.

---

Our representative visited the Royal Free Hospital this week, and was greatly impressed by the good order and taste, and by the attractive courtesy extended to visitors by every member of the nursing staff. As an experienced matron she rejoiced to find this atmosphere, which is sometimes lacking in hospitals of importance where every visitor has a right to expect the most courteous treatment. If manners make the man, it is infinitely more true that a good manner is one of the most important assets a woman can possess in regard to her work and also in her social relations. Courtesy costs nothing, but the impression it produces and its lasting effects upon recipients have a value which it would be difficult to over-estimate. So Miss Cox-Davies and her staff of workers may be congratulated and thanked that the reception visitors meet with when at the Royal Free Hospital makes them eloquent in their praise of the working staff.

---

We recently gave an account of the accommodation for warriors at the Royal Free Hospital. Here, again, throughout each section of the accommodation provided for 140 patients there is evidence of the presence of a directing mind, administrative gifts of a high order, and a taste in decoration and equipment which many visitors have envied. At the first blush most of us would hold that the British officer would prefer to have a ward to himself. At the Royal Free, however, the out-patient hall has been converted into one large ward for officers, and presents a most attractive appearance to the eye, whilst its acceptability to officers seems to be undoubted from the fact that at least two generals have been patients in this ward. The equipment of the dining-rooms, the thoughtful service of meals at small tables, with accommodation for four officers at each, the recreation room, and, indeed, the whole of the arrangements present features worthy of imitation. One such point which struck our representative was the plan which provided for the keeping and polishing of the floors and corridors under contract by Ronuk, Limited. This arrangement has given infinite satisfaction and produced most satisfactory results. We should be glad to have invitations from matrons for our representative to visit their institutions that due testimony may be borne to any special arrangements to which the matron may attach importance, and generally.

---

A letter read at the last meeting of the managers of the Hartlepools Hospital from the matron, Miss Stevenson, intimated that she had been promoted to the post of matron in the war hospital at Sheffield where she has been serving for over a year. As the pay of this position exceeds that which she formerly had at the Hartlepools Hospital, the managers of the latter will no longer be asked to make up her salary to the level at which it stood before the war broke out. Miss Stevenson added that her new appointment is only temporary, during the war, and that she hopes to return to her old post when peace is declared. The facts disclosed in this letter are distinctly to the credit of the Hartlepools Hospital, as well as to Miss Stevenson. It is creditable if a like generosity on the part of institutions to their matrons and sisters is the rule throughout the country. Unfortunately, many hospitals are themselves in such narrowed circumstances through the war that they might find it a serious tax on their resources both to pay a substitute and also to supplement to its pre-war figure the income of a matron or sister away on war duty.

---

*** It is our invariable practice to pay for all items of news which we may publish. The minimum payment is 5s. Paragraphs of about twenty lines in length—that is to say, of 150 words or less—are preferred. In this section during the war we propose to give 10s. to the correspondent who may send us in any week the best incident connected with hospital life and work. In this way we hope to afford to all practical aid and encouragement; while those who reciprocate will be able to render us invaluable service. Contributions should be addressed to The Editor, The Hospital, 28 and 29 Southampton Street, Strand, London, W.C., and, to be in time for the current week's issue, should reach him by the first post on Mondays.

For Matrons' and Sisters' Appointments see page 336. For Review under head of "British Nursing" see page 328.

